# Influence of initial occlusal severity on time and efficiency of Class I malocclusion treatment carried out with and without premolar extractions

**DOI:** 10.1590/2176-9451.19.4.038-049.oar

**Published:** 2014

**Authors:** Ruben Leon-Salazar, Guilherme Janson, José Fernando Castanha Henriques, Vladimir Leon-Salazar

**Affiliations:** 1 Masters student in Orthodontics, School of Dentistry — USP/ Bauru.; 2 Full professor, School of Dentistry — USP/ Bauru.; 3 PhD resident in TMD and Orofacial Pain, School of Dentistry — University of Minnesota.

**Keywords:** Class I malocclusion, Efficiency, Time, Tooth extraction

## Abstract

**Introduction:**

The aim of this retrospective study was to compare the occlusal outcomes, duration and efficiency of Class I malocclusion treatment carried out with and without premolar extractions in patients with different degrees of initial malocclusion severity.

**Methods:**

Complete records of 111 patients were obtained and divided into two groups: Group 1 consisted of 65 patients at an initial mean age of 13.82 years old treated with four premolar extractions; whereas Group 2 consisted of 46 patients at an initial mean age of 14.01 years old treated without extractions. Two subgroups were obtained from each group (1A, 1B, 2A and 2B) with different degrees of malocclusion severity according to the initial values of PAR index. Compatibility was assessed using chi-square and t-tests. The subgroups were compared by means of Analysis of Variance (ANOVA).The variables that might be related to treatment duration and efficiency were assessed using the multiple linear regression analysis.

**Results:**

Initial malocclusion severity was positively related to the amount of occlusal correction and consequently to a higher efficiency index. Moreover, extraction protocol showed a positive relationship with treatment duration and a negative relationship with treatment efficiency.

**Conclusion:**

Extraction and non-extraction protocols for correction of Class I malocclusion provide similar satisfactory results; however, the extraction protocol increases the overall treatment duration. Orthodontic treatment is more efficient in cases with high initial malocclusion severity treated with a non-extraction protocol.

## Abstract (pt)

**Introdução:**

o objetivo desse estudo retrospectivo foi comparar os resultados oclusais, o tempo e o grau de eficiência do tratamento da má oclusão de Classe I realizado com e sem extrações em pacientes que apresentavam diferentes tipos de severidade oclusal inicial.

**Métodos:**

a amostra foi composta pelas documentações de 111 pacientes, divididas em dois grupos: Grupo 1 (n = 65), com idade inicial média de 13,82 anos, tratados com extrações; Grupo 2 (n = 46), com idade inicial média de 14,01 anos, tratados sem extrações. De cada grupo, foram obtidos dois subgrupos (1A, 1B, 2A e 2B) com severidades oclusais diferentes (alta e baixa), de acordo aos valores iniciais do índice PAR. A avaliação da compatibilidade foi realizada por meio do teste qui-quadrado e do teste *t*. Os subgrupos foram comparados por meio da análise de variância (ANOVA) e foi realizada a análise de regressão linear múltipla para avaliação das variáveis que poderiam estar relacionadas com o tempo e com a eficiência do tratamento.

**Resultados:**

a severidade oclusal inicial esteve diretamente relacionada à quantidade de sua correção e, consequentemente, à obtenção de um maior índice de eficiência; por outro lado, a utilização do protocolo de extrações de pré-molares mostrou uma relação direta com o tempo de tratamento e inversa com a eficiência do tratamento.

**Conclusão:**

no tratamento da má oclusão de Classe I, podem ser obtidos resultados oclusais satisfatórios com uma maior quantidade de correção das alterações oclusais nos casos com maior severidade inicial, e um maior tempo de tratamento quando o tratamento envolve extrações dentárias.

## INTRODUCTION

Assessing treatment outcomes by means of occlusal indexes allows us to understand the effects different types of appliances, techniques and treatment protocols produce on dental occlusion,^1,4,8,13,20,28,33^ treatment time^2,3,10,21,34^ and efficiency. In this context, efficiency is described as the achievement of the best results within a shorter period of time.^19,31^

Some authors have observed the influence of dental extractions on correction of initial malocclusion severity, showing better occlusal results when a non-extraction protocol was used.^6^ However, they observed that in Class II malocclusion cases, the protocol that included the extraction of two maxillary premolars yielded better occlusal outcomes than the non-extraction and the four-premolar extraction protocols.^19,20^

Regarding treatment time, the literature generally highlights dental extractions as one of the main factors for increased treatment time.^6,10,34^ Contrary to those findings, Beckwith et al^3^ stated that the difference in treatment time between extraction and non-extraction protocols is not significant. Other authors also assessed the influence of malocclusion severity on treatment time and found no relation between treatment duration and initial malocclusion severity.^16^. Nevertheless, other studies have shown that there is a direct correlation between initial malocclusion severity and the treatment duration.^6,10^

Unfortunately, these previous studies used mixed samples that included different types of malocclusions and treatment protocols. Therefore, the applicability of their findings is limited and cannot be extrapolated to Class I malocclusion.

The objective of this study was to compare the occlusal outcomes, treatment duration and efficiency of two different protocols for Class I malocclusion: non-extraction and four-premolar extractions, in order to elucidate the effects of dental extractions on orthodontic treatment performed for this specific malocclusion.

## MATERIAL AND METHODS

### Material

The sample of this retrospective study comprised patients with Class I malocclusion and similar pre-treatment characteristics who were treated with four premolars extractions or without extractions. Patients were selected from the Master's and Postgraduate Orthodontic programs at the School of Dentistry of University of São Paulo, Bauru, Brazil. In selecting the sample, the following inclusion criteria were applied:

» Class I malocclusion treated without extractions or with extraction of four premolars, two maxillary and two mandibular.
» Presence of all permanent teeth up to the first molar.
» Presence of crowding not greater than 8 mm.
» Absence of supernumerary teeth.
» Absence of impacted teeth.
» Absence of abnormalities in tooth size and / or shape.
» Treatment with full fixed Edgewise appliances.
» No history of orthognathic surgery.
» Full orthodontic records available for review.

The sample comprised the initial and final orthodontic records of 111 patients who were divided into two groups according to the extraction protocol used as part of the orthodontic treatment.

Group 1 consisted of 65 patients, 24 males (36.92%) and 41 females (63.08%), with initial mean age of 13.82 years old (ranging from 10.69 to 22.04 years), who had Class I malocclusion and were treated with extraction of four premolars, two maxillary and two mandibular (Tables 4 and 5).

**Table 4:** Compatibility of groups.

Sex	GROUP 1 (Extraction) n = 65	GROUP 2 (Non-extraction) n = 46	Total
Females	41	30	71
Males	24	16	40
Total	65	46	111
X^2^ = 0.535	GL = 1	p = 0.817	

**Table 5:** Comparison of the initial characteristics using t-test.

VARIABLES	GROUP 1 (Extraction) n = 65	GROUP 2 (Non-extraction) n = 46	DF	p
	Mean ± SD	Mean ± SD		
PARi	19.92 ± 8.08	17.89 ± 6.96	109	0.170
CROWDING	4.94 ± 1.59	4.32 ± 1.87	109	0.065
AGE	13.82 ± 2.11	14.01 ± 1.78	109	0.620

Group 2 consisted of 46 patients treated without extractions, 16 males (34.78%) and 30 females (65.22%) with initial mean age of 14.01 years old (ranging from 11.04 to 21.54 years) (Tables 4 and 5). Both groups were treated with full fixed appliances using the simplified Edgewise technique.

Since previous studies have shown that severity of malocclusion could influence the treatment duration,^6,10^ we further divided each group, based on their initial occlusal index, into two subgroups with different malocclusion severity (high and low). Thus, the four subgroups, two in each group, with high and low initial malocclusion severity had the following characteristics (Table 6).

**Table 6:** Results of ANOVA and Tukey's test regarding the initial characteristics of subgroups 1A, 1B, 2A e 2B. Subgroups were classified according to their initial malocclusion severity.

Variables	GROUP 1 (Extraction)		GROUP 2 (Non-extraction)		ANOVA	
	Subgroup 1A High Severity n = 22	Subgroup 1B Low Severity n =22	Subgroup 2A High Severity n =15	Subgroup 2B Low Severity n = 15		
	Mean ± SD	Mean ± SD	Mean ± SD	Mean ± SD	F	p
PARi	29.09^a^ ± 5.32	11.68^b^ ± 2.40	25.60^a^ ± 5.15	10.87^b^ ± 2.97	97.28	0.000*
CROWDING	5.17 ± 1.67	4.64 ± 1.62	4.08 ± 2.39	4.19 ± 1.72	1.35	0.265
AGE	13.54 ± 2.18	13.34 ± 1.25	13.88 ± 1.06	14.09 ± 1.45	0.79	0.504

* Statistically significant: P < 0.05.

Subgroup 1A ( High severity; n = 22) comprised 8 males and 14 females with initial mean age of 13.54 ± 2.18 years (minimum 10.69, maximum 21.25). Subgroup 1B ( Low severity; n = 22) comprised 6 males and 16 females with a initial mean age of 13.34 ± 1.25 years (minimum 11.15, maximum 15.53). Subgroup 2A ( High severity; n = 15) comprised 5 males and 10 females with a initial mean age of 13.88 ± 1.06 years (minimum 11.90, maximum 15.82). Subgroup 2B (Low severity; n =15) comprised 4 males and 11 females with a initial mean age of 14.09 ± 1.45 years (minimum 11.40, maximum 15.99).

### Methods

#### Clinical records

Patients' orthodontic records were used to obtain the demographic and clinical information included in the analysis: sex, date of birth, age at treatment onset, proposed treatment protocol, including extraction and non-extraction of premolars, and length of active orthodontic treatment.

To estimate total treatment time, the starting date was defined as the date when placement of first molar bands or first direct bonding occurred, whereas final date was defined as the date when orthodontic retainers were delivered.

#### Dental cast analysis

##### Assessment of mandibular crowding

The amount of mandibular crowding was calculated based on the difference between the arch perimeter (circumference measured from the mesial of one permanent first molar to its antimere) and the sum of the mesio-distal width of all mandibular permanent teeth except molars.^26^

##### Calculation of occlusal index

The occlusal index was calculated according to the weighted Peer Assessment Rating (PAR index) advocated by DeGuzman et al^9^ which includes the assessment of five occlusal features (posterior occlusion, overjet, overbite, midline and maxillary tooth displacements) with well-defined measurement criteria (Table 1).

**Table 1:** Criteria applied to score each component of PAR index^9^.

	Occlusal relationships	Discrepancy	Score	Weight
**POSTERIOR OCCLUSION**	Anteroposterior	Good interdigitation – Class I, II or III	0	2
		Less than half of premolar width	1	
		Half of premolar width	2	
	Vertical	No discrepancy in intercuspation	0	2
		Posterior open bite on at least two teeth greater than 2 mm	1	
	Transverse	No cross-bite	0	2
		Cross-bite tendency	1	
		Single tooth in cross-bite	2	
		More than one tooth in cross-bite	3	
		More than one tooth in scissor bite	4	
***OVERJET***	Positive	0 - 3 mm	0	5
		3.1 - 5 mm	1	
		5.1 - 7 mm	2	
		7.1 - 9 mm	3	
		Greater than 9 mm	4	
	Negative	No discrepancy	0	5
		One or more teeth edge-to-edge	1	
		One single tooth in cross-bite	2	
		Two teeth in cross-bite	3	
		More than two teeth in cross-bite	4	
***OVERBITE***	Negative	No open bite	0	3
		Open bite less than and equal to 1 mm	1	
		Open bite 1.1 - 2 mm	2	
		Open bite 2.1 - 3 mm	3	
		Open bite greater than or equal to 4 mm	4	
	Positive	Less than or equal to 1/3 coverage of lower incisor	0	3
		Greater than 1/3. but less than 2/3 coverage of lower incisor	1	
		Greater than 2/3 coverage of lower incisor	2	
		Greater than or equal to full coverage of lower incisor	3	
**DISPLACEMENT**	Crowding Spacing Impaction	0 - 1 mm displacement	0	1
		1.1 - 2 mm displacement	1	
		2.1 - 4 mm displacement	2	
		4.1 - 8 mm displacement	3	
		Greater than 8 mm	4	
		Impacted teeth	5	
Midline		Coincident and up to ¼ lower incisor width	0	3
		Deviated ¼ to ½ lower incisor width	1	
		Deviated more than ½ lower incisor width	2	

The scores for PAR index calculation^30^ are recorded according to the following:

1. Posterior occlusion.

Posterior occlusion, also described as "buccal segment relationship" in the original PAR index, comprises the zone from the distal anatomical contact point of canine to the mesial anatomical contact point of first permanent molar. Posterior dental relationship is assessed in three planes of space and scores are given to anteroposterior, vertical and transverse discrepancies according to Table 1. These scores are added and the final value is multiplied by two. Each posterior segment, right and left, is recorded separately.

2. Overjet.

Positive or negative overjet is recorded using the most prominent surface of any central or lateral incisors as reference. During this measurement, the ruler is held parallel to the occlusal plane and radial to the line of the arch. The magnitude of the overjet is transformed into a score according to Table 1 and then multiplied by 5.

3. Overbite.

Overbite is recorded as the proportion of the lower incisor crown that is covered by upper incisors or the amount of open bite, in millimeters, taking as reference the tooth with greater overlap. The score obtained according to Table 1 is then multiplied by 3.

4. Midline.

Discrepancy of maxillary midline is assessed in relation to lower central incisors using the score in Table 1 which is then multiplied by 3.

5. Maxillary tooth displacement.

Displacements such as crowding, spacing and impacted teeth are recorded in the maxillary anterior region, only. These occlusal features are recorded considering the shortest distance between contact points of adjacent teeth parallel to the occlusal plane. These measurements are transformed into scores and added according to the criteria defined in Table 1. A tooth is considered impacted when the space available for this tooth is less than 4 mm.

We calculated the PAR index for each of the pretreatment and post treatment dental casts (n = 222) using the criteria described above and using the scores specified in Table 1. PAR index was termed initial PAR (PARi) when obtained from the pretreatment models, and final PAR (PARf) when calculated in post-treatment casts. The higher the numerical value obtained in these indexes, the more severe the malocclusion, because PAR index is obtained by applying scores to the intra-arch (e.g. crowding) and inter-arch (e.g. overbite, overjet, crossbite, midline) dental relationships as well as by using an ordinal scale starting at 0 for a normal value. All measurements in the initial and final casts were obtained using a digital caliper (Mitutoyo, Kawasaki, Japan) with accuracy closed to 0.1 mm.

#### Assessing changes in occlusal discrepancy

Changes in occlusal discrepancy produced by each treatment protocol were calculated by subtracting PARf from PARi values (PARi - PARF). The numerical reduction in the index accounted for occlusal changes directly related to treatment protocol.^29,30^ In addition, the percentage of PAR reduction (PcPAR) during treatment was calculated to verify the amount of improvement produced in relation to the initial severity of malocclusion.^29,30^

For this calculation, we applied the following mathematical formula:

$$\mathrm{PcPAR}=\frac{\mathrm{PARi}-\mathrm{PARf}}{\mathrm{PARi}}\times 100$$

#### Treatment efficiency index (TE)

Treatment efficiency was defined as the greatest occlusal index change produced within the shortest treatment time. It was calculated using the following formula, in which the denominator is the total treatment time expressed in months:^19,31^

$$T_{E}=\frac{\mathrm{PcPAR}}{\mathrm{TIME}}$$

### Statistical analysis

Errors of the method were assessed by repeating the measurements on 20 initial and 20 final dental casts randomly selected from the sample. Repeated measurements were taken approximately one month after the first occlusal index calculation (Table 3). The formula proposed by Dahlberg^7^ (S^2^ = Σd^2^/2n) was applied to estimate random errors, while paired t-test was used to analyze systematic errors.^18^

**Table 3:** Results of systematic and random errors assessed using depended t-test and Dahlberg's formula.

Variables	1^st^ Measurement (n = 20)	2^nd^ Measurement (n = 20)	gl	p	Dahlberg
	Mean ± SD	Mean ± SD			
PARi	19.25 ± 6.07	19.40 ± 6.28	19	0.527	0.72
PARf	5.00 ± 3.15	5.25 ± 3.31	19	0.234	0.65

Initial compatibility regarding gender distribution between the two study groups was assessed using the non-parametric chi-square test (Table 4). T-test was also used to assess other baseline characteristics, such as age, malocclusion severity, and amount of mandibular crowding (Table 5). Subgroups 1A, 1B, 2A and 2B were compared using Analysis of Variance (ANOVA). Tukey's test was used to investigate the hypothesis that severity of PARi influences the treatment duration (Tables 6 and 7). Multiple linear regression was used to assess the influence of initial malocclusion severity, mandibular crowding and the extraction/non-extraction protocols over treatment efficiency (Tables 8 and 9). All statistical analyses were performed using Statistica software. P value ≤ 0.05 was considered significant.

**Table 7:** Comparison of occlusal changes, treatment duration and treatment efficiency between subgroups 1A, 1B, 2A e 2B.

Variables	GROUP 1 (Extraction)		GROUP 2 (Non-extraction)		ANOVA	
	Subgroup 1A High Severity N = 22	Subgroup 1B Low Severity N =22	Subgroup 2A High Severity N =15	Subgroup 2B Low Severity N = 1		
	Mean ± SD	Mean ± SD	Mean ± SD	Mean ± SD	F	p
PARf	5.95 ± 3.93	5.36 ± 3.92	4.33 ± 3.90	5.53 ± 3.44	0.55	0.652
PARi-PARf	23.14^a^ ± 5.05	6.32^b^ ± 3.82	21.27^a^ ± 7.70	5.33^b^ ± 4.34	60.90	0.000*
PC-PAR	80.00^a^ ± 12.54	54.81^b^ ± 32.26	81.36^a^ ± 17.91	45.74^b^ ± 40.70	7.40	0.000*
TIME	24.84^a^ ± 4.18	24.57^a^ ± 7.33	20.39^ab^ ± 8.15	18.24^b^ ± 7.24	4.09	0.010*
IET-PAR	3.32^ab^ ± 0.83	2.42^a^ ± 1.67	4.37^b^ ± 1.38	3.31^ab^ ± 3.22	3.24	0.027*

* * Statistically significant: P < 0.05.

**Table 8:** Multiple regression analysis using treatment duration as a dependent variable

Variables	Coefficient	SE	t	p
(Constant)	22.748	2.657	8.562	0.0000
PARi	0.031	0.087	0.344	0.7316
CROWDING	0.080	0.389	0.870	0.3864
PROTOCOL	0.332	1.369	-3.593	0.0005*

SE: standard error

R^2^= 0.1297. Length of treatment=22.75 + 0.33(Protocol) + 0.080(Crowding) + 0.031 (PARi)

* Protocol: 0 - Non-extraction; 1 - Extraction.

**Table 9:** Multiple regression analysis using treatment efficiency as a dependent variable

Variables	Coefficient	SE	t	p
(Constant)	2.075	0.639	3.247	0.0016
PARi	0.238	0.021	2.589	0.0110*
CROWDING	-0.057	0.094	-0.615	0.5397
PROTOCOL	-0.261	0.329	2.808	0.0059*

R^2^=0,1149. Treatment efficiency = 2.075 - 0.261(Protocol) - 0.057(Crowding) + 0.238 (PARi)

* Protocol: 0 - Non-extraction; 1 - Extraction.

## RESULTS

No systematic errors^18^ were found for repeated measurements one month after the initial assessment. Random errors^7^ were considered negligible (Table 3).

Groups were compatible regarding age, sex, mandibular crowding and PARi (Tables 4 and 5). As shown in Table 6, malocclusion severity was significantly different between subgroups with high PARi (1A, 2A) and low PARi (1B, 2B) severity. The difference between PARi was of approximately 16 points. Subgroups were compatible in all other variables.

Final occlusal outcome, assessed by means of PARf, was similar in all subgroups (Table 7). However, numerical and proportional reduction in the occlusal index was significantly greater in subgroups with high PARi (1A, 2A) than in subgroups with low PARi (2A, 2B). The treatment duration for the non-extraction subgroups was about four (2A) to six (2B) months less than the extraction subgroups; however, treatment time was only significantly reduced in subgroup 2B that started with a low PARi. Treatment was also more efficient in the group with high malocclusion severity treated without extractions (Table 7).

Multiple linear regression analysis showed that of the three variables evaluated (PARi, CROWDING and PROTOCOL), only the treatment protocol with extractions showed significant positive correlation with treatment duration (Table 8). Regarding treatment efficiency, initial malocclusion severity showed a positive influence on the efficiency index, while treatment protocol influenced it negatively (Table 9).

## DISCUSSION

### Sample and compatibility

The overall objective of this study was to compare two different treatment protocols for Class I malocclusion. For this reason, the sample only included patients with Class I malocclusion who were treated with or without extraction of four premolars. We focused on this specific type of malocclusion because the compatibility of groups regarding initial malocclusion severity decreased the risk of bias. As showed in previous studies, treatment length and efficiency varies according to the amount of initial anteroposterior discrepancy.^6,19,31,34^ Distribution of sex, age, PARi, and mandibular crowding were also compatible between groups, which reduced the risk of confounding and selection bias.

During preliminary data collection, we realized that most patients treated without extractions had mandibular crowding of 8 mm or less, while cases treated with extractions showed greater amount of crowding. Therefore, we only included patients with initial mandibular crowding not greater than 8 mm in order to eliminate the influence of this variable on the results of our study.^32^

Cases that had their initial treatment plan changed during the course of treatment (e.g. non-extraction cases that ended up with extractions) were excluded from the study to avoid the influence of this factor on treatment duration.^21^

The aforementioned inclusion and exclusion criteria were applied to 4000 clinical charts belonging to the Department of Orthodontics' archives of the School of Dentistry - University of São Paulo/Bauru. A sample of 111 subjects was obtained. Considering that the incidence of Angle Class I malocclusion is of approximately 55 %,^15^ we expected to come up with a larger study sample. However, the meticulous application of these criteria resulted in the elimination of a large number of potential participants with Class I malocclusion. The study sample reduced even further because some of the clinical charts did not have the orthodontic documentation that met the specific needs of this study.

## METHODS

We used the PAR occlusal index to quantify both, pre-treatment discrepancy and post-treatment occlusal outcomes, since its accuracy and reliability has been previously validated. Moreover, the PAR index is an objective and user friendly analysis method that has been extensively used in similar studies.^1,4,8,9,13,19,30^ Besides allowing us to compare our findings with previous studies, the use of PAR index allowed us to investigate the compatibility of both groups regarding the severity of initial malocclusion,^4,9,19,29,30^ as well as the numerical and the percentage of improvement obtained in each group at the end of treatment. We associated the percentage of occlusal improvement with treatment duration in order to obtain an index capable of objectively expressing the degree of treatment efficiency (T_E_), as previously described by other studies.^19,31^

Lack of significant systematic errors and reduced value of random errors found in this study reflect standardization and accuracy of measurements (Table 3) and are related to the calibration of the examiner prior to data collection. The simple and objective assessment of dental casts by means of PAR index also allowed us to obtain a high degree of precision and reproducibility.

## COMPARISON BETWEEN GROUPS AND VARIABLES

### Post-treatment occlusal outcomes

The comparison of PARf values between subgroups showed that final occlusal relationships in all subgroups were similarly satisfactory (Table 7). PARf index of non-extraction and extraction groups ranged from 4.33 to 5.95, thus showing a good occlusal outcome in all patients regardless of severity of their initial occlusal discrepancy. These values ​​were similar to those found in prior studies conducted with Class I patients. For instance, Birkeland et al^4^ found PARf values of 5.9 and 6.2 for cases treated with and without extractions, respectively. Likewise, other authors, such as Willems et al,^38^ Freitas et al,^8,13^ found pooled PARf values of 5.1, 6.32, and 5.65, respectively. However, these studies used different criteria for sample selection. Birkeland et al^4^ included cases treated with fixed appliances in both maxillary and mandibular arches and cases treated with fixed appliances in a single arch. Willems et al^38^ included patients treated with removable orthodontic or functional orthopedic appliances and cases treated with fixed appliances in one or two dental arches. Freitas et al^8,13^ only included patients treated with extraction of four premolars and fixed appliances in both dental arches.

Several other studies have assessed the amount of correction of initial occlusal discrepancy using PAR index. Nevertheless, these studies included different types of malocclusion in their samples because their main objectives were to audit the quality of orthodontic treatment provided and the factors that may influence treatment duration and efficiency provided in private practice, university-based and hospital-based clinics. Some of these studies reported ​similar ​PARf values,^6,12,17,24^ while others showed higher PARf^11,12,24,33^ and lower PARf values than our study.^25,28,31,39^ The difference in samples and methodology prevented us to make further comparison with these studies.

The high PARf values found in some previous studies were related to cases treated with removable functional orthopedic appliances,^11,12,24,33^or cases receiving treatment for only one of the dental arches, or the use of historical samples that do not show results as good as recently treated cases.^11^ Additionally, the experience of the treatment provider was also found to be directly associated with the quality of occlusal outcomes.^11,12,28,31,33^ Moreover, the common characteristic among studies that showed low PARf values was that orthodontic treatment was provided by a certified specialist, regardless of being a private or public setting.

### Treatment-related occlusal improvement

The amount of occlusal improvement, measured as the numerical and percentage reduction in PAR index, was significantly greater in subgroups with high initial malocclusion severity (1A and 2A) than in subgroups with low initial malocclusion severity (1B and 2B) (Table 6). The direct correlation between the amount of initial occlusal discrepancy and the amount of occlusal improvement has been previously reported.^4,6,12,13,24,27,33^ The higher the initial occlusal discrepancy, the greater reduction in PARf and the greater percentage of occlusal improvement.

For instance, Robb et al^31^ observed percentages of PAR index reduction of 84.5% in adults and 88.1% in adolescents while Woods, Lee and Crawford,^39^ found 82.2% and 87.2% of occlusal improvement in patients treated with and without extractions, respectively. These high levels of occlusal improvement could be attributed to the inclusion of patients with high PARi (24.9 to 26.6) treated by specialist in private practices.^11,12,28,33^

### Treatment duration

To analyze treatment duration, we took into account initial malocclusion severity (high and low) and differences between subgroups regarding age, sex, mandibular crowding, and dentition. Our results showed that patients treated without extractions had overall shorter treatment than those treated with extraction of four premolars. However, difference was significant only in the non-extraction subgroup with low severity (Table 7). As shown in the multiple regression analysis and in agreement with previous studies,^6,10,34^ extractions had a direct incremental effect on the length of treatment (Table 8). Our results corroborate the conclusions by Turbill, Richmond and Wrigh,^34^ indicating that treatment with extractions in Class I malocclusion has an additional phase (closure of extraction spaces) as compared to treatment without extractions, thus resulting in increased total treatment time.

Previous studies have shown that there is a direct influence of initial malocclusion severity over treatment duration, meaning that severe malocclusions require longer treatment time,^6,10^. Our findings, however, are similar to Grewe and Hermanson's^16^ results: we did not find a significant correlation between initial malocclusion severity and treatment duration.

Total treatment time found in our study was similar to several other studies investigating Class I malocclusion. For instance, Wes Fleming et al^37^ reported a treatment time of 20.6 ± 6 months for non-extraction cases, whereas Freitas et al^8,13^ and Nakamura^23^ reported treatment times of 24.96, 25.08 and 28.95 months, respectively, for patients treated with extractions. Kocadereli^22^ found greater treatment time for non-extraction and extraction cases, 26.35 ±13.25 months and 31.53 ± 14.10 months, respectively. Interestingly, Germec and Taner^14^ found that borderline Class I cases treated with extraction lasted 24.8 ±6.9 months while those treated with stripping lasted 17 ± 4 6 months. On the other hand, Skidmore et al^32^ reported that treatment duration of their Class I sample was 21.9 months, despite the fact that they used different treatment protocols. Minor variations in treatment time between studies are probably related to differences in methodology and sample.

### Treatment efficiency

Treatment efficiency is defined as the satisfactory occlusal relationship obtained within the shortest treatment time, assuming that the outcomes meet clinician's and patient's expectations. Treatment efficiency index allowed us to objectively assess and compare the degree of efficiency of the two protocols used in this study.

Results showed a higher efficiency ratio (4.37) for the subgroup with high malocclusion severity treated without extractions and a lower efficiency ratio (2.42) for the subgroup with low severity treated with extractions (Table 7), while the two other subgroups showed an intermediate value (3.3). These findings were mainly due to the following factors:

#### Initial malocclusion severity

Similarly to previous studies,^4,6,12,13,24,27,33^ our results revealed a direct relationship between initial malocclusion severity and its correction, as analyzed numerically and in percentage (Table 7). Thus, knowing that the percentage of correction (PcPAR) has a direct relationship with efficiency, it would be expected that initial severity also influence it, as shown by multiple regression analysis (Table 9). Therefore, treatment efficiency was positively influenced by high initial occlusal discrepancy (subgroups 1A and 2A) and negatively influenced by low initial severity (subgroups 1B and 2B).

#### Treatment duration

While the occlusal changes resulting from treatment have a proportional relationship with efficiency ratio, treatment duration showed an inversely proportional relationship.^19,31^ Thus, the lower values ​​in the length of treatment in subgroups treated without extractions resulted in higher values for the efficiency ratio.

#### Treatment protocol

The multiple regression analysis showed a direct relationship between the extraction protocol and longer treatment time (Table 8), and an inverse relationship with the efficiency ratio (Table 9). This result was expected, as several studies have also shown a direct relationship between the number of extractions and a longer treatment time,^6,10,20,34^ which suggests that extractions negatively influence treatment efficiency.

Therefore, the significant greater efficiency found in the subgroup with high PARi values treated without extractions (subgroup 2A) was mainly due to the positive influence of a high value of initial severity and treatment protocol. An opposite effect was observed in the subgroup with low PARi treated with extractions (subgroup 1B), which showed a low efficiency index.

### Clinical considerations

Extraction of permanent teeth for orthodontic purposes has been used for a long time.^5,36^ However, the controversy surrounding its use is far from being resolved. The popularity of extraction and non-extraction protocols have alternated in orthodontic history,^5,36^ showing a "pendulum" effect, i.e., favoring one protocol for a period of time and then the other in the next period. New appliances and techniques have also influenced the use of tooth extraction as part of the orthodontic treatment (e.g. cephalometry, expanders, distalization, brackets, archwire alloys).^36^ Currently, the search for better esthetic, functional and stable results has decreased this discussion, and extractions are more accepted as means and not as objectives of orthodontic treatment. Its use has also decreased and it is only considered after careful evaluation of all factors involved in each particular case.^19,20,22,35^

In this study, we found that initial malocclusion severity did not significantly influence the duration of orthodontic treatment. However, initial severity was directly related to the amount of its correction and, as a consequence, to a higher degree of efficiency, which corroborates the results reported in previous studies.^4,6,12,13,24,27,33^

Extraction of premolars as part of Class I treatment showed a direct relationship with treatment duration and an inverse relationship with treatment efficiency. This positive relationship between the extraction of premolars and treatment duration had already been observed in other studies;^6,10,34^ however, the high heterogeneity of the methodology, sample, types of malocclusion, and appliances used limited the application of their results to specific situations, such as treatment of Class I malocclusion. Moreover, Beckwith et al^3^ showed that there was no relationship between extractions and an increased treatment time, making it difficult to generalize these conflicting results.

The treatment objectives regarding the occlusal outcomes in all subgroups were the same (tooth alignment, ideal overjet and overbite, and maintenance of Class I molar relationship). Therefore, the main difference between groups was whether or not their treatment included extraction of four premolars. The greater treatment time in the extraction group could be explained by the need for an additional phase that involved closure of the extraction space by retraction of maxillary and mandibular anterior teeth. The size of the remaining extraction space depended on the amount of initial crowding.^34^

This study confirms the positive influence of initial malocclusion severity on treatment efficiency and the negative influence of dental extractions on orthodontic treatment duration. Clinicians can expect satisfactory occlusal outcomes with a greater amount of correction in cases with severe occlusal discrepancy and a longer treatment time when it involves dental extractions. Our findings can be used to inform patients and parents about the expected treatment time for correction of Class I malocclusion. Additionally, it can be used to calculate professional fees.

## CONCLUSIONS

The methodology and results of this study led us to the following conclusions:

1. Occlusal outcomes were satisfactory and similar in the four subgroups evaluated, regardless of the protocol (extraction or non-extraction) used.
2. Initial malocclusion severity showed a significant direct relationship with the amount of occlusal improvement and with the efficiency ratio; but no influence on orthodontic treatment duration.
3. Extraction of premolars for treatment of Class I showed a direct relationship with treatment duration and an inverse relationship with treatment efficiency.

## Figures and Tables

**Table 2:** Description of variables used.

ABBREVIATIONS	DESCRIPTION
PARi	Initial PAR index
APINH	Initial amount of mandibular crowding
AGE	Age at the beginning of treatment
PARf	Final PAR index
PARi-PARf	Improvement of occlusal discrepancy
PC-PAR	Improvement of occlusal discrepancy (percentage)
Time	Treatment duration in months
IET-PAR	Treatment Efficiency Index
